# Divergent Nutrient Resorption Strategies in C4 Desert Shrubs: Stoichiometric Evidence From Assimilative Branches

**DOI:** 10.1002/ece3.72853

**Published:** 2026-01-12

**Authors:** Su‐Su Wei, Yuan‐Yuan Zhang, Xin‐Yue Jin, Yue Zhang, Xiao‐Bing Zhou, Gang Huang, Ye Tao

**Affiliations:** ^1^ College of Life Sciences Shihezi University Shihezi China; ^2^ State Key Laboratory of Ecological Safety and Sustainable Development in Arid Lands, Xinjiang Institute of Ecology and Geography, Chinese Academy of Sciences Xinjiang China; ^3^ China‐Tajikistan Belt and Road Joint Laboratory on Biodiversity Conservation and Sustainable Use, Xinjiang Institute of Ecology and Geography, Chinese Academy of Sciences Urumqi China; ^4^ Xinjiang Key Laboratory of Biodiversity Conservation and Application in Arid Lands, Xinjiang Institute of Ecology and Geography, Chinese Academy of Sciences Xinjiang China

**Keywords:** assimilative branch, desert shrub, influencing factor, nutrient limitation, nutrient resorption efficiency

## Abstract

*Calligonum* species are widely distributed across the arid desert belt stretching from North Africa to Central Asia. Their leaves are nearly fully degraded, and photosynthetic activity is predominantly undertaken by assimilative branches (ABs). Remarkably, *Calligonum* represents the only known lineage within the family Polygonaceae to exhibit the C4 photosynthetic pathway. To date, on the regional scale, the nutrient resorption patterns of ABs of different *Calligonum* species are still unclear. We investigated three representative species from distinct taxonomic sections native to the Junggar Desert of northwestern China: *C. mongolicum* (CM; Sect. Medusa), *C. leucocladum* (CL; Sect. Pterococcus), and *C. junceum* (CJ; Sect. Calliphysa). Green ABs and AB litters were collected during the summer and autumn, respectively, to assess interspecific differences in nitrogen (N), phosphorus (P), and potassium (K) resorption efficiencies (NRE, PRE, and KRE, respectively), and explore their stoichiometric relationships, variation patterns, and the environmental influences. Across all species, the nutrient resorption efficiencies (NuREs) followed the order: KRE (65.03% ± 0.57%) > PRE (53.57% ± 0.48%) > NRE (23.36% ± 0.70%). Among the three taxa, CM exhibited the highest NRE (29.20% ± 1.24%) and PRE (62.44% ± 0.45%), whereas KRE was lowest in CJ (57.41% ± 1.41%). All three species exhibited a scaling relationship between NRE and PRE with slope > 2, indicating that N was resorbed more rapidly than P. The scaling relationship of PRE–KRE showed considerable interspecific variation, with CJ exhibiting a negative slope (−0.492). NuREs were positively correlated with nutrient concentrations in summer green ABs but negatively correlated with those in AB litters. Within species, the three NuREs generally exhibited similar patterns of variation across geographic, climatic, and edaphic gradients, yet marked interspecific differences persisted. Soil and climatic conditions were identified as the primary environmental determinants of NuRE variability, although species‐specific responses indicated that different nutrient elements were affected by distinct interactions among environmental factors. In summary, the three *Calligonum* species demonstrated differentiated nutrient resorption strategies, closely tied to both their internal nutrient status and environmental contexts. These findings provide valuable insights into nutrient use strategies and adaptive mechanisms in *Calligonum* and other assimilative‐branch shrubs inhabiting arid ecosystems.

## Graphical abstract

We assessed interspecific differences in nutrient resorption efficiencies (N, P, K) of assimilative branches in three *Calligonum* species from the Junggar Desert. The results revealed distinct nutrient resorption strategies and scaling relationships among species, closely linked to both internal nutrient status and environmental factors. These findings highlight adaptive mechanisms of *Calligonum* in arid ecosystems.

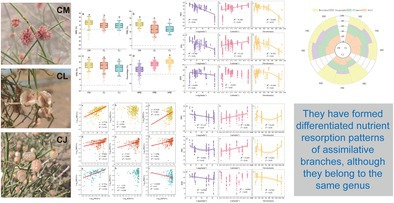

## 1 Introduction

The theory of stoichiometry holds significant theoretical value for advancing the understanding of plant physiological and ecological processes, biogeochemical cycles, and ecosystem functioning. Among its core topics, nutrient resorption plays a critical role. Nutrient resorption refers to the process by which nutrients are withdrawn from senescing tissues and reallocated to other parts of the plant. This mechanism, shaped by long‐term evolutionary processes, represents an essential nutrient conservation strategy that is vital for plant growth and reproduction (Aerts 1996). By enhancing nutrient use efficiency, nutrient resorption enables plants to maximize the utility of nutrients already acquired from the soil, thereby ensuring a continued nutrient supply for subsequent growth phases (Killingbeck 1996; Vergutz et al. 2012). As a result, the ability of plants to thrive in nutrient‐poor environments may not solely depend on their capacity to absorb nutrients from the soil, but also on the efficiency of internal nutrient recycling through resorption processes (Aerts and de Caluwe 1997). Nutrient resorption is influenced by a wide range of factors. Key drivers include latitude, climatic conditions (such as mean annual precipitation and temperature), soil properties, and vegetation types. In addition to environmental variables, phylogenetic lineage may also play a role in shaping nutrient resorption traits; however, environmental factors—particularly climate—often explain a greater proportion of the observed variation than phylogenetic constraints (Zhang et al. 2012). Furthermore, nutrient resorption is closely linked to a plant's internal nutrient status. The leaf nutrient resorption efficiency is associated with nutrient concentrations in both mature and senescing leaves and is regulated by the plant's nutrient limitation status (Sun et al. 2023; Zhang et al. 2015). In summary, nutrient resorption enhances plant adaptability in nutrient‐deficient or resource‐limited environments, serving as a key strategy for improving plant competitiveness, productivity, and resistance to environmental stress (Veneklaas et al. 2012).

Nitrogen (N), phosphorus (P), and potassium (K) are essential nutrients for plants and are classified as mobile elements. Global analyses of terrestrial vegetation indicate that more than 50% of foliar N and P can be resorbed by plants during leaf senescence (Veneklaas et al. 2012; Yuan et al. 2005; Aerts and Chapin 2000), and others have compared nutrient resorption patterns between deciduous and evergreen shrubs, finding that deciduous species generally exhibit higher N and P resorption efficiencies (NRE and PRE, respectively) in their leaves. Deciduous plants tend to increase N and P concentrations in their leaves during a limited growing season to achieve faster growth rates. Even under relatively nutrient‐rich conditions, they often resorb nutrients at the end of the growing season to minimize nutrient loss (Reich and Oleksyn 2004; He et al. 2020). Arid desert ecosystems represent a critical component of the global terrestrial biosphere, characterized by low precipitation, high evaporation rates, sparse vegetation cover, and nutrient‐poor soils, all of which contribute to their fragile ecological stability (Whitford and Wade 2002; Xiao et al. 2014). Plants inhabiting these extreme environments often exhibit specialized morphological and physiological adaptations, such as the reduction or complete loss of leaves and the use of assimilative branches (ABs; hereafter) as the primary photosynthetic organs. Studies have shown that desert plants tend to have relatively low NuREs and limited nutrient recovery potential, with drought stress being a likely factor constraining their capacity to reclaim nutrients from senescing tissues (Li et al. 2022; Yuan et al. 2005). However, patterns in nutrient resorption can still be discerned in desert plants. For example, P limitation in arid soils is often more severe than N limitation, leading to a generally higher PRE than NRE among desert species (Luo et al. 2020). Therefore, elucidating the nutrient utilization strategies of various desert plant groups is essential for a deeper understanding of their survival mechanisms and offers valuable guidance for the conservation and utilization of genetic resources in arid regions.

Plants of the genus *Calligonum* (belonging to family Polygonaceae, and the only C4‐plants in this family) are typical desert shrubs distributed across arid regions from Asia to north Africa. As an evolutionarily ancient genus, *Calligonum* species also serve as important dominant taxa in the desert ecosystems of Central Asia (Zhang and Mao 1989). Based on fruit morphological traits, *Calligonum* has been taxonomically classified into four sections: Sect. *Calliphysa* (inflated fruits), Sect. *Calligonum* (basal‐winged fruits), Sect. *Medusa* (spiny fruits), and Sect. *Pterococcus* (winged fruits). *Calligonum* species are well adapted to extreme desert conditions, exhibiting high tolerance to heat and nutrient‐poor soils. They are fast‐growing, easily propagated, and capable of withstanding wind erosion and sand burial, making them highly resilient in shifting sand environments (Feng et al. 2008). These properties enable *Calligonum* to establish pioneer communities for sand stabilization (Xie et al. 2014). In addition, several species are valued as forage plants, being particularly favored by camels and sheep. Their population renewal strategies are diverse and include clonal reproduction, which enhances their capacity to survive in harsh arid habitats (Yu et al. 2010, 2001). Previous studies have reported physiological variations both within the same *Calligonum* species across different environments and among different species inhabiting similar environments (Pan et al. 2017; Zhao et al. 2014). For instance, a common garden experiment conducted at the Turpan Desert Botanical Garden revealed significant interspecific differences in nutrient concentrations among ABs, older stems, and reproductive organs of three introduced *Calligonum* species (Liu et al. 2024). At present, studies on nutrient resorption in *Calligonum* species are extremely limited. In two fixed‐site studies conducted in the northern deserts of Xinjiang, nutrient resorption in ABs of *Calligonum mongolicum* followed the pattern PRE > NRE > K resorption efficiency (KRE) (Luo et al. 2020). However, to date, little is known about the interspecific differences, correlations, and spatial patterns of NREs in ABs of *Calligonum* species at broader regional scales.

What are the patterns of variation in NuREs among different *Calligonum* species? And what are the primary factors driving these differences? To address this gap, we selected three *Calligonum* species from distinct taxonomic sections that are widely distributed across the Junggar Desert: *Calligonum mongolicum* (CM; Sect. *Medusa*), *Calligonum leucocladum* (CL; Sect. *Pterococcus*), and *Calligonum junceum* (CJ; Sect. *Calliphysa*). NuREs were calculated based on nutrient concentrations in ABs during the peak growing season and in the corresponding senesced AB litters. The objectives of this study were threefold: (1) to examine interspecific differences in NuREs among the three *Calligonum* species; (2) to explore allometric relationships among NRE, PRE, and KRE; and (3) to investigate the spatial variation of NuREs in relation to geographic, climatic, and edaphic factors and identify their primary environmental drivers. Drawing on stoichiometry theory and previous findings from both desert and non‐desert plant studies, we hypothesize that despite taxonomic relatedness, the nutrient resorption traits and their spatial patterns will differ significantly among species. These differences are expected to follow general ecological hypotheses such as the Power Law of Nutrient‐to‐Nutrient Scaling, Nutrient‐Limited Operational Strategies, and Climate‐Driven Hypothesis (Rajpurohit and Eiteman 2022). The results of this study will contribute to a deeper understanding of nutrient‐use strategies and adaptive mechanisms in *Calligonum* and other shrubs with assimilative‐branches. Moreover, the findings will also provide valuable empirical evidence for improving biogeochemical cycling models in arid ecosystems and inform future conservation and utilization of desert plant resources.

## 2 Study Area and Methods

### 2.1 Study Area

The study area is located in the Junggar Desert in northern Xinjiang, China (34°09′–49°08′ N, 73°25′–96°24′ E). The desert is surrounded by four major mountain ranges (the Tianshan, Beita, and Altai Mountains, along with the western highlands of the Junggar Basin); it represents a typical temperate inland desert in Central Asia. The Junggar Desert is distinguished by its arid climate, characterized by low annual precipitation (50–200 mm), low relative humidity (average below 45%), high evaporation (> 2000 mm), ample solar radiation, and pronounced diurnal and annual temperature fluctuations (Zhang 2002). Situated in the south‐central part of the Junggar Desert, the Gurbantunggut Desert is the only semi‐fixed and fixed desert in China influenced by cold and moist air masses originating from the Atlantic Ocean. Its plant community composition and species richness differ significantly from other deserts in China. Dominant plants include *Haloxylon ammodendron*, *Haloxylon persicum*, and small shrubs such as *Ephedra przewalskii* and *Artemisia songarica*, along with abundant ephemeral and ephemeroid species that are uncommon in other temperate deserts of the country (Zhang and Mao 1989). Moreover, biological soil crusts (BSCs) at various successional stages are extensively distributed across the region. These BSCs represent a crucial biological component for maintaining the stability of desert surfaces (Zhang et al. 2010).

### 2.2 Sample Collection and Processing

#### 2.2.1 Collection of Plant and Soil Samples

**FIGURE 1:**
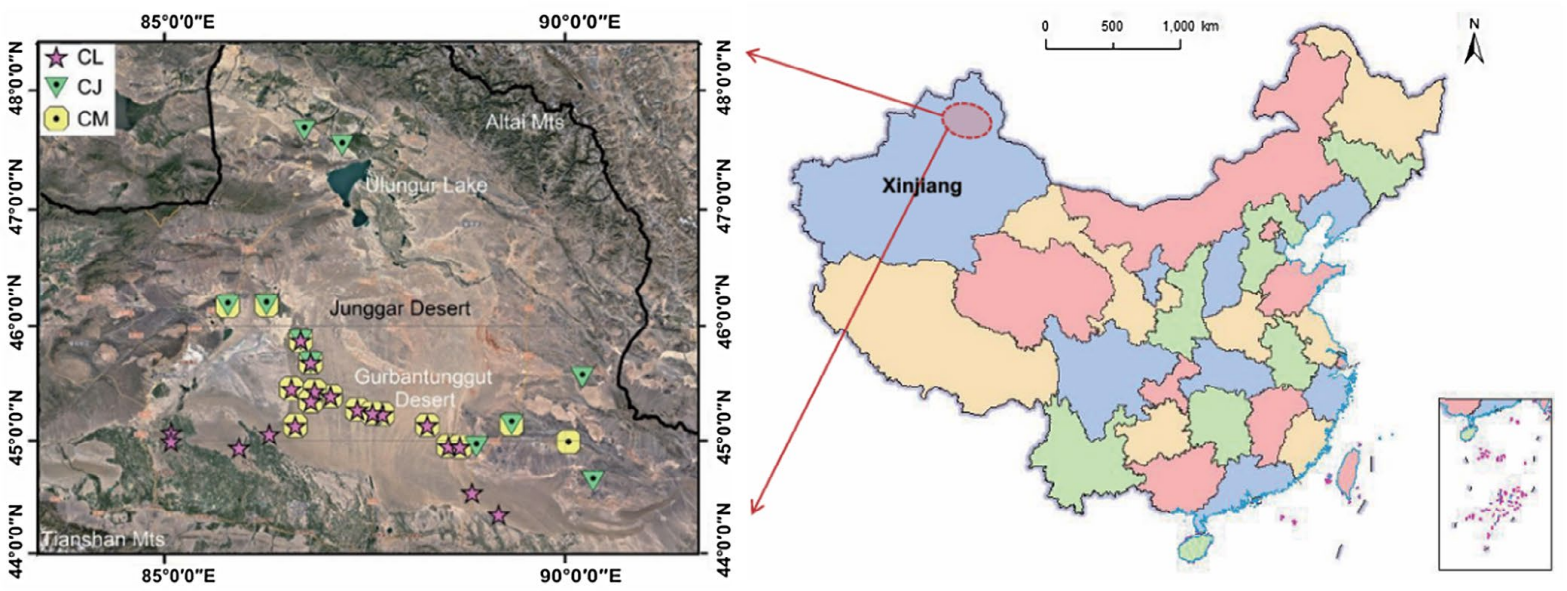
Distribution of sampling sites for three *Calligonum* species. CJ, *C.* junceum; CL, *C. leucocladum* ; CM, *C. mongolicum* . The red elliptical marker on the map of China indicates the location of the Junggar Basin in Xinjiang.

The first field investigation was conducted in June 2022 during the fruiting period of *Calligonum* species. Based on long‐term survey data, sampling plots were established at known *Calligonum* species distribution sites across the Junggar Desert at a regional scale. In total, 28 sampling sites were surveyed, including 17 CM plots, 19 CL plots, and 10 CJ plots (in the northmost two plots, no litters were collected); some plots harbored coexisting species (Figure 1). Each plot was 30 m × 30 m in size. Within each plot, individuals of CM, CL, or CJ were randomly selected, and 30 similarly sized individuals were tagged. In cases where fewer than 30 individuals were present, all available individuals within the plot were marked. A total of 904 plants were tagged across all plots. During the summer, green ABs were first collected from the upper canopy of mature branches. For each sampling individual, 8–10 g of fresh ABs was harvested, placed in labeled envelopes, and stored in insulated boxes with ice packs for prompt transport to the laboratory. Subsequently, litter collection nets were set up beneath each tagged plant. In November 2022, AB litters were collected from each labeled plant in each sampling plot. Litter samples, weighing 8–10 g per plant, were placed in labeled envelopes and quickly transported back to the laboratory for further processing.

Each sampling site was georeferenced using a GPS device to record the precise longitude, latitude, and altitude. At each sampling plot, three replicate mixed soil samples (0–10 cm depth; five‐point sampling method) were randomly collected. The soil samples were placed in labeled zip bags and transported back to the laboratory, where they were air‐dried in a shaded environment.

#### 2.2.2 Processing of Plant and Soil Samples

Plant samples were oven‐dried at 70°C until constant weight. After manual sorting, samples were ground using a disc vibratory mill (RS200; Retsch, Germany), sealed in zip bags, and stored in a dry, dark environment until analysis. Before chemical analysis, the powdered material was sieved and homogenized. N concentration (mg g^−1^) in ABs was measured using an elemental analyzer (Multi N/C 3100; Analytik Jena AG, Jena, Germany). P concentration (mg g^−1^) was measured using the molybdenum–antimony anti‐spectrophotometric method. K concentration (mg g^−1^) was measured by flame spectrophotometry (FP640; Jingke Co., Shanghai, China). The stoichiometric ratios (N:P:K) were calculated based on mass ratios.

Soil samples were air‐dried in a shaded environment and passed through a 2 mm sieve before analysis. Ten soil physicochemical parameters were measured: soil organic matter (SOM, g kg^−1^), total nitrogen (TN, g kg^−1^), total phosphorus (TP, g kg^−1^), total potassium (TK, g kg^−1^), available nitrogen (AN, mg kg^−1^), available phosphorus (AP, mg kg^−1^), available potassium (AK, mg kg^−1^), soil pH, electrical conductivity (EC, μS cm^−1^), and mean particle diameter (M_Z_, mm). The mixed soil samples were sequentially sieved, and the M_Z_ was calculated using GRADISTAT software. All other soil variables were analyzed following the standard methods described by Bao (2000).

#### 2.2.3 Acquisition of Meteorological Data

Environmental variables were obtained from publicly available databases following the method described by Zhang, Tao, et al. (2022), and were used to analyze the factors influencing NuREs. Mean annual precipitation (MAP), mean annual temperature (MAT), solar radiation (Srad), soil water content (SWC), potential evapotranspiration (PET), and wind speed (VS) data were sourced from the National Qingzang Plateau Data Center (Peng 2020, 2022). Aridity was defined as 1 − AI, where AI is defined as the ratio of MAP to PET. In this study, aridity was used as a key climatic indicator.

### 2.3 Statistical Analyses

#### 2.3.1 Calculation of Nutrient Resorption Efficiency

Nutrient resorption efficiency (NuRE) was calculated by matching nutrient concentrations in green ABs during the peak growing season and their corresponding litter from the same tagged individuals. Among various available methods, this study adopted a mass‐based formula (Vergutz et al. 2012):

(1) $$\text{NuRE}=\left({\mathrm{Nu}}_{\mathrm{gr}}–{\mathrm{Nu}}_{\mathrm{se}}\times \text{MLCF}\right)/N_{\mathrm{gr}}\times 100\%,$$

where NuRE refers to nutrient resorption efficiency, including NRE, PRE, and KRE. Nu_gr_ is the nutrient concentration in green ABs at the peak growing period (summer), and Nu_se_ is the nutrient concentration in senesced ABs (autumn litters). MLCF is the mass loss correction factor; in this study, MLCF was set at 0.832 (Zhang, Luo, et al. 2022). Three stoichiometric ratios of NuREs were also calculated.

Before statistical analysis, data were tested for normality. One‐way ANOVA was performed to assess differences in NuREs both within and among species. Levene's test was used to evaluate the homogeneity of variances. When the assumption of homogeneity was met, multiple comparisons were conducted using Duncan's multiple range test; when variances were unequal, Tamhane's T2 test was applied instead. All figures were generated using Origin 2021 (OriginLab Corporation, USA) and R version 4.4.1.

#### 2.3.2 NuREs in ABs and the Relationships With Nutrient Concentrations in Green and Senesced Tissues

The scaling relationships among NRE, PRE, and KRE were represented by a power function: *Y* = *β X* ^*α*^, where *Y* and *X* denoted the contents of any two elements; *β* was a constant, and *α* was the scaling exponent. Typically, the power function underwent logarithmic transformation before fitting. When *α* = 1, it signified an isometric relationship, whereas *α* ≠ 1 indicated an allometric relationship (with *α* > 1 representing a hyperallometric relationship and *α* < 1 indicating a hypoallometric relationship). At the same time, we examined whether significant allometric relationships exist between NuREs and nutrient concentrations in green ABs and litters for the three *Calligonum* species. Data analysis was performed using the “SMATR.”

#### 2.3.3 Analysis of Influencing Factors

To examine the geographic trends in NuREs and stoichiometric ratios of the three *Calligonum* species, both linear and nonlinear regression models were applied to evaluate their variation patterns along gradients of longitude, latitude, altitude, MAP, and aridity. Pearson correlation analysis was subsequently performed to investigate the relationships between environmental variables and the stoichiometric parameters of green ABs, litters, and NuREs across the three species. The R packages “rdacca.hp” and “UpSetVP” were employed to quantitatively assess the relative contributions of environmental factors to the stoichiometric traits of ABs. The “rdacca.hp” package was used to conduct canonical redundancy analysis (RDA) and hierarchical partitioning, which allowed us to quantify the relative importance of three groups of environmental variables: climatic variables (MAT, MAP, Srad, VS, PET, aridity), soil properties (SOC, TN, TP, TK, AN, AP, AK, pH, EC, MZ, SWC), and geographic factors (longitude, latitude, altitude). The results of variance partitioning and hierarchical decomposition were visualized using UpSet matrix generated with the “UpSetVP” package. These plots distinguish between the unique effects and common effects. The bar charts on the upper part of the UpSet matrix plot displayed the variance partitioning results explained by unique or common effects of variables, with the number indicating the percentage of variance for the corresponding explanatory variable. Data analysis was performed using R 4.4.1 (R Development Core Team 2024), and the final UpSet matrix visualizations were created using the UpSetView function in TBtools.

## 3 Results

### 3.1 Nutrient Resorption Characteristics of N, P, and K in ABs of Three *Calligonum* Species

**FIGURE 2:**
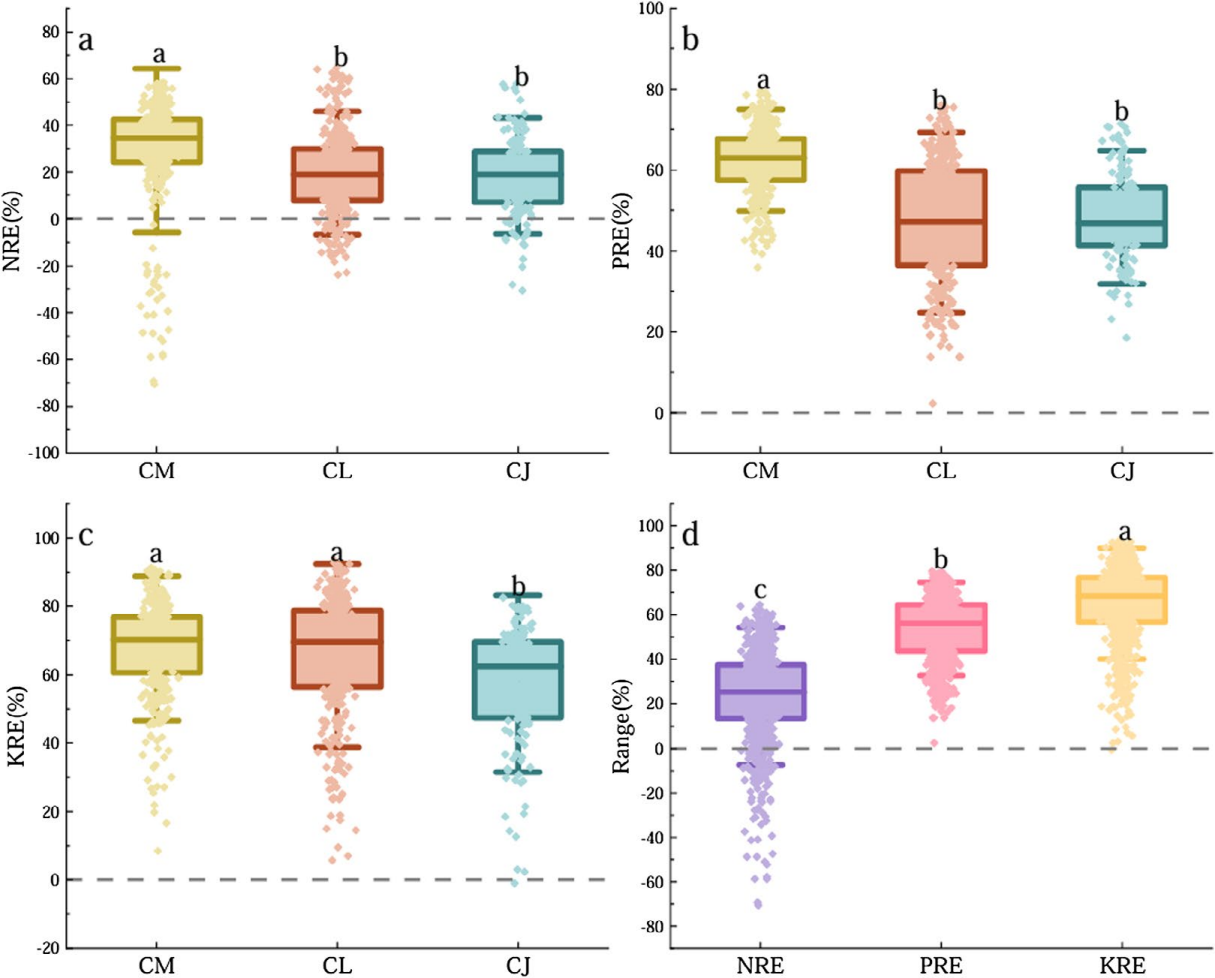
Differences in NRE, PRE, and KRE of ABs of three *Calligonum* species. Different lowercase letters indicate significant differences (*p* < 0.05). CJ, *C.* junceum; CL, *C. leucocladum* ; CM, *C. mongolicum* .

Regarding NRE, there was no significant difference between CL (19.68% ± 0.93%) and CJ (18.35% ± 1.34%), both of which were significantly lower than that of CM (29.20% ± 1.24%) (Figure 2). Similarly, PRE did not differ significantly between CL (47.07% ± 0.79%) and CJ (48.26% ± 0.89%), but both were significantly (*p* < 0.05) lower than that of CM (62.44% ± 0.45%). For KRE, CM (67.68% ± 0.75%) and CL (65.57% ± 0.96%) exhibited higher values, while CJ (57.41% ± 1.41%) showed significantly (*p* < 0.05) lower KRE. Overall, CM exhibited the highest NRE, PRE, and KRE among the three species. When comparing mean values across species, significant differences (*p* < 0.05) were observed among the three NuREs, with KRE being the highest (65.03% ± 0.57%), followed by PRE (53.57% ± 0.48%), and NRE being the lowest (23.36% ± 0.70%).

### 3.2 NuREs in ABs of Three *Calligonum* Species and the Allometric Relationships With Nutrients in Green and Senesced Tissues

**FIGURE 3:**
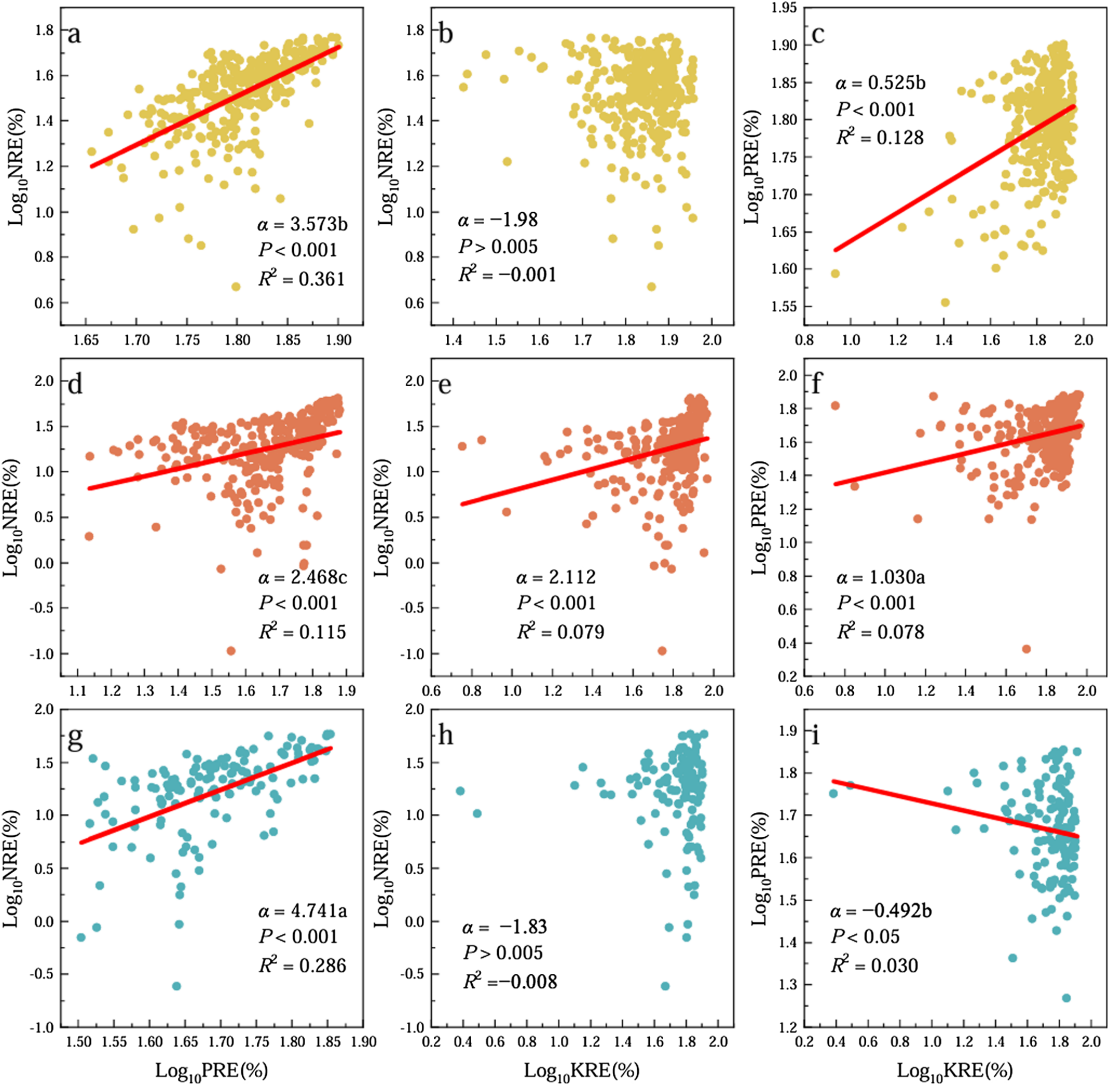
Allometric relationships among NRE, PRE, and KRE in ABs of three *Calligonum* species. Different lowercase letters indicated that the scaling slopes (*α*) were significantly different among different species (*p* < 0.05). CM, *C. mongolicum* (a–c); CL. *C. leucocladum* (d–f); CJ, *C. junceum* (g–i).

Among the different *Calligonum* species, the hyperallometric relationships (slope *α* > 1) were observed between NRE and PRE of all species (Figure 3), with scaling exponents ranked as follows: CJ (*α* = 4.741) > CM (*α* = 3.573) > CL (*α* = 2.468) (*p* < 0.05). For NRE–KRE, only CL exhibited a significant hyperallometric relationship (*p* < 0.05), with a scaling exponent of *α* = 2.112. The PRE–KRE relationship showed contrasting patterns among species: in CM, it followed a hypoallometric scaling with *α* = 0.525; in CL, it conformed to isometric scaling (*α* = 1.030); while in CJ, the PRE was negatively correlated with KRE and exhibited a scaling exponent of *α* = −0.492. As such, the three species exhibited completely different allometric relationships among NuREs.

For all three *Calligonum* species, NuREs showed significantly (*p* < 0.05) positive allometric relationships with nutrient concentrations in green ABs in summer, with no common slope observed among species (Table 1). Specifically, NREs exhibited allometric relationships with Ngr for all species (*α* > 1), with scaling exponents ranked as follows: CL (*α* = 5.151) ≈ CJ (*α* = 4.545) > CM (*α* = 2.646). The PRE–Pgr relationship showed contrasting patterns among species: CM exhibited a hypoallometric relationship (*α* = 0.859), whereas both CL (*α* = 2.240) and CJ (*α* = 1.309) displayed significant hyperallometric relationships, with CL having the steepest slope. For the KRE–Kgr relationship, CJ showed the highest scaling exponent (*α* = 1.510; hyperallometric); by contrast, both CM (*α* = 1.028) and CL (*α* = 0.989) exhibited isometric relationships (*α* ≈ 1), suggesting a proportional change in KRE with respect to K concentration in green ABs.

**TABLE 1:** Allometric relationships between NuREs and nutrient concentrations of green ABs of three *Calligonum* species.

*Y*	*X*	Species	Allometric scaling exponent (*a*)				Isometric test	
			*R* ^2^	*p*	*a*	95% CI	*F*	*p*
NRE	N_gr_	CM	0.092	0.000	2.646b	2.384–2.937	456.239	0.000
		CL	0.363	0.000	5.151a	4.708–5.635	2939.618	0.000
		CJ	0.277	0.000	4.545a	3.930–5.256	860.241	0.000
		Common slope	—	—	—	—	—	—
PRE	P_gr_	CM	0.229	0.000	0.859c	0.783–0.942	10.613	0.000
		CL	0.274	0.000	2.240a	2.049–2.448	392.093	0.000
		CJ	0.503	0.000	1.309b	1.169–1.465	22.541	0.000
		Common slope	—	—	—	—	—	—
KRE	K_gr_	CM	0.401	0.000	1.028b	0.949–1.115	0.463	0.497
		CL	0.484	0.000	0.989b	0.917–1.067	0.079	0.779
		CJ	0.456	0.000	1.510a	1.339–1.704	48.333	0.000
		Common slope	—	—	—	—	—	—

*Note:* Different lowercase letters indicated that the scaling slopes (*α*) were significantly different among different species (*p* < 0.05). N_gr_, P_gr_, and K_gr_ indicated N, P, and K concentrations in green ABs, respectively.

Abbreviations: CJ, *C.* junceum; CL, *C. leucocladum* ; CM, *C. mongolicum* .

For all three species, NuREs exhibited significantly (*p* < 0.05) negative allometric relationships with litter nutrient concentrations, with no common slope detected among species (Table 2). Specifically, the NRE–Nse scaling exponents ranked as follows: CM (*α* = −1.943) > CJ (*α* = −5.172) ≈ CL (*α* = −4.819), with no significant difference in scaling exponents between the latter two species. For the PRE–P_se_, the scaling exponents ranked as follows: CM (*α* = −0.703) > CL (*α* = −1.668) ≈ CJ (*α* = −1.569), with no significant difference in scaling slopes between the latter two species. In the case of KRE–K_se_, scaling exponents ranked as follows: CM (*α* = −0.799) > CL (*α* = −1.091) > CJ (*α* = −2.159); significant differences were detected among the three species (*p* < 0.05). The KRE–K_se_ scaling exponent indicated a stronger negative response in CJ compared to the other species.

**TABLE 2:** Allometric relationships between NuREs and litter nutrient concentrations of three *Calligonum* species.

*Y*	*X*	Species	Allometric scaling exponent (*a*)			
			*R* ^2^	*p*	*a*	95% CI
NRE	N_se_	CM	0.358	0.000	−1.943b	−2.122, −1.780
		CL	0.177	0.000	−4.819a	−5.337, −4.352
		CJ	0.055	0.006	−5.172a	−6.105, −4.381
		Common slope	—	—	—	—
PRE	P_se_	CM	0.469	0.000	−0.703b	−0.759, −0.651
		CL	0.585	0.000	−1.668a	−1.784, −1.560
		CJ	0.309	0.000	−1.569a	−1.793, −1.373
		Common slope	—	—	—	—
KRE	K_se_	CM	0.379	0.000	−0.799c	−0.868, −0.736
		CL	0.220	0.000	−1.091b	−1.198, −0.994
		CJ	0.142	0.000	−2.159a	−2.510, −1.857
		Common slope	—	—	—	—

*Note:* Different lowercase letters indicated that the scaling slopes (*α*) were significantly different among different species (*p* < 0.05). N_se_, P_se_, and K_se_ indicated N, P, and K concentrations in litters, respectively.

Abbreviations: CJ, *C. junceum* ; CL, *C. leucocladum* ; CM, *C. mongolicum* .

### 3.3 Change Patterns of NRE, PRE, and KRE of Three *Calligonum* Species Along With Environment Variables

**FIGURE 4:**
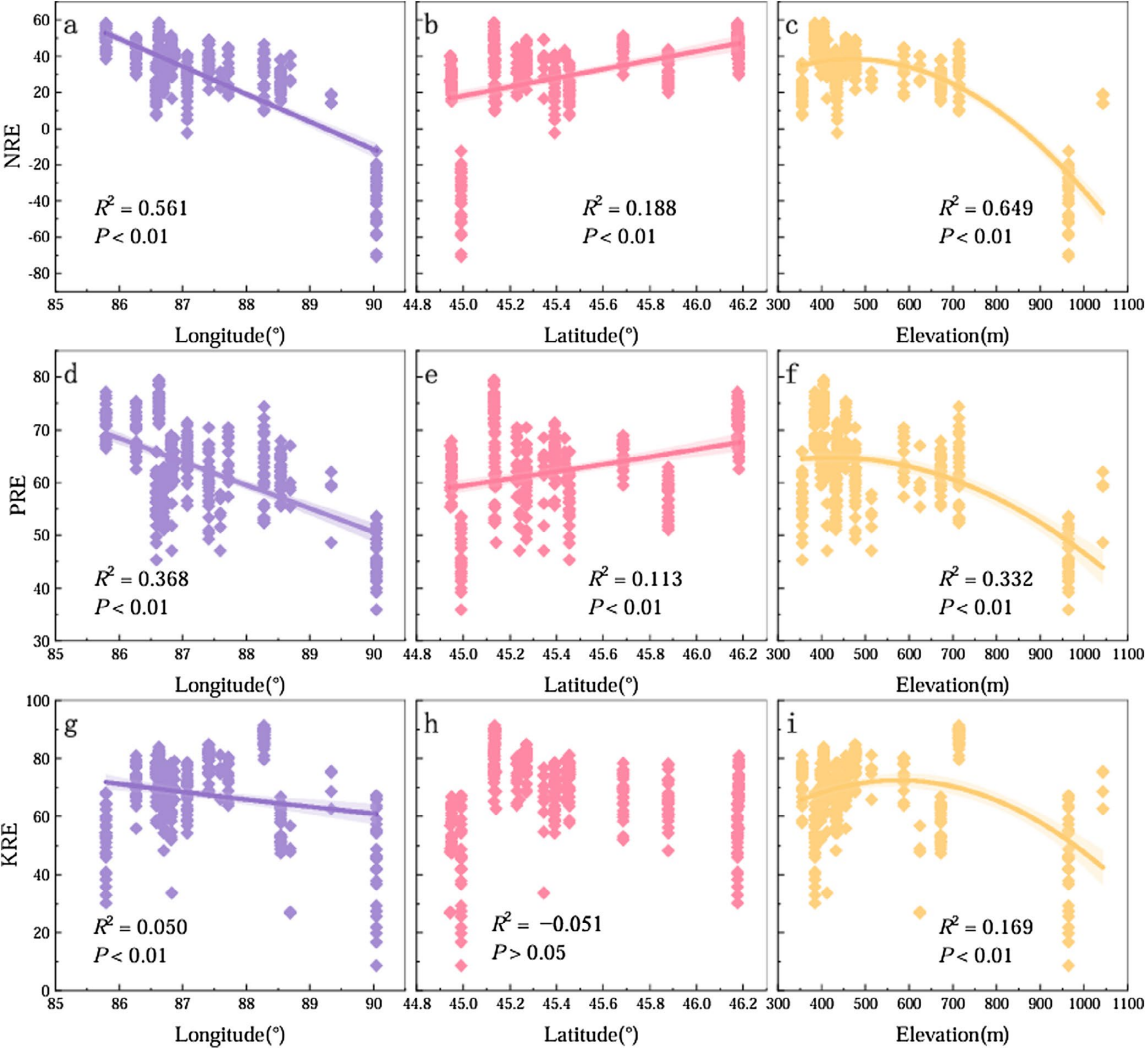
Variation patterns of NRE, PRE, and KRE of ABs of *C. mongolicum* along with longitude, latitude, and altitude.

For CM, the geographic variation patterns of NRE and PRE in ABs were consistent across longitude, latitude, and altitude gradients (Figure 4). NRE and PRE decreased progressively with increasing longitude, increased with increasing latitude, and exhibited a nonlinear pattern with altitude—showing a slight decline initially, followed by a rapid decrease at higher altitudes. KRE in CM also declined with increasing longitude, but showed no clear pattern along the latitudinal gradient. With increasing altitude, KRE first increased and then declined. Overall, NRE, PRE, and KRE in CM tended to decline with increasing longitude and altitude.

**FIGURE 5:**
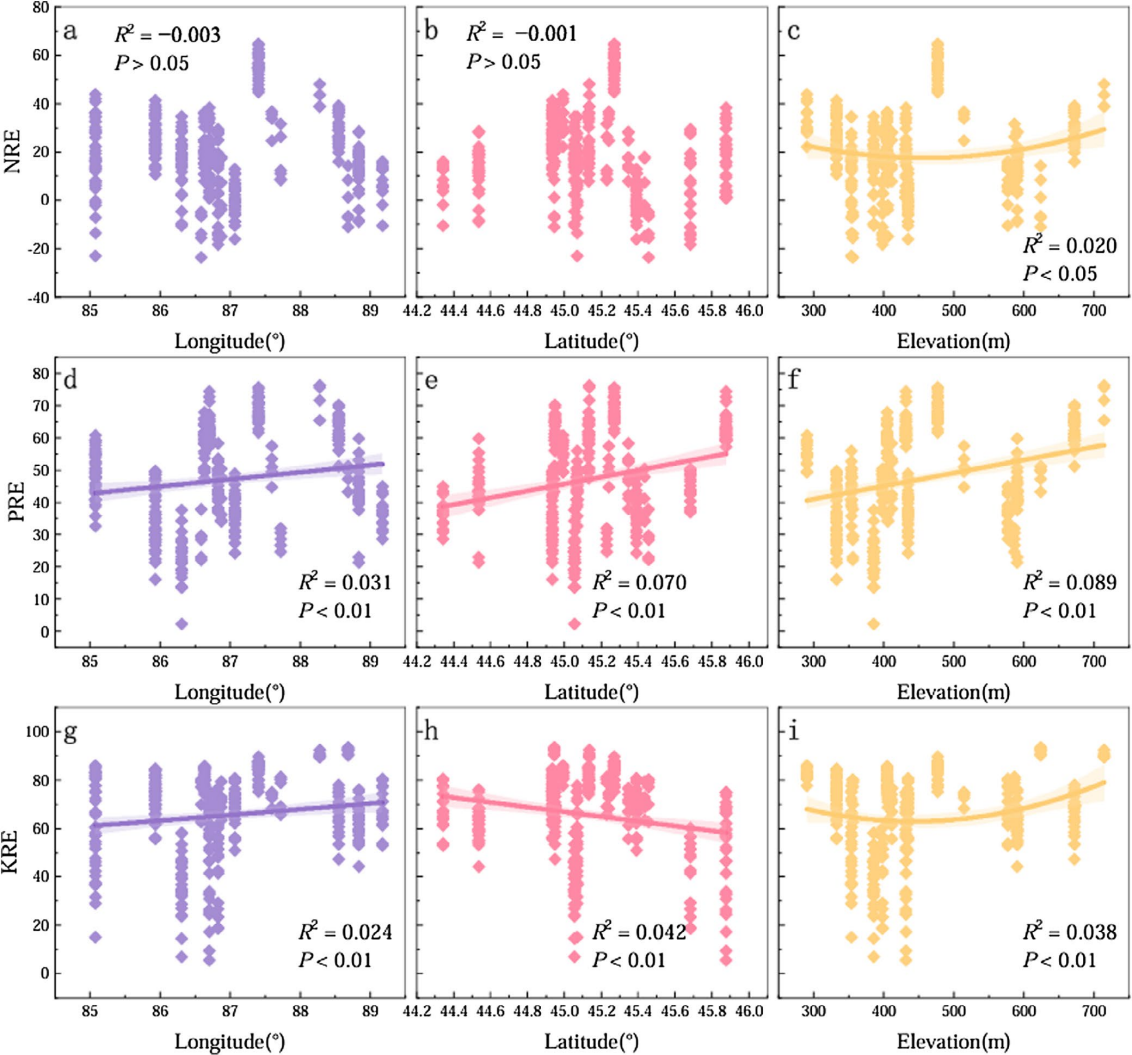
Variation patterns of NRE, PRE, and KRE of ABs of *C. leucocladum* along with longitude, latitude, and altitude.

For CL, the NRE in ABs showed no significant variation pattern along longitude or latitude gradients, while along the altitude gradient, NRE first decreased and then increased (Figure 5). PRE in CL increased progressively with rising longitude, latitude, and altitude. In contrast, KRE showed a gradual increase with increasing longitude, a decreasing trend with latitude, and a unimodal pattern along the altitude gradient—first declining, then increasing at higher altitudes.

**FIGURE 6:**
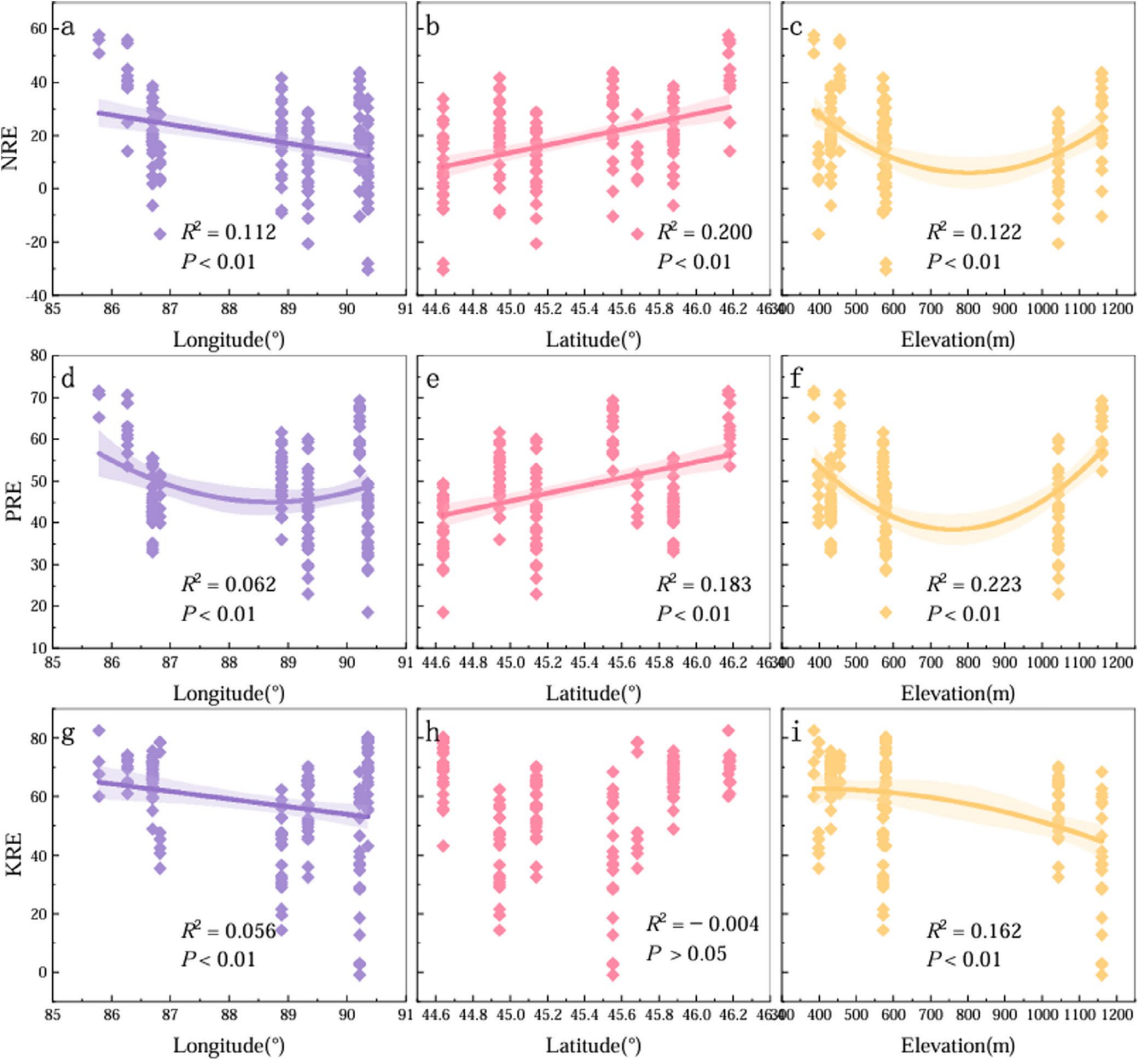
Variation patterns of NRE, PRE, and KRE of ABs of *C. junceum* along with longitude, latitude, and altitude.

For CJ, the geographic variation patterns of NRE and PRE in ABs were consistent across longitude, latitude, and altitude gradients (Figure 6). Both NRE and PRE gradually decreased with increasing longitude, increased with increasing latitude, and exhibited a nonlinear trend with altitude—initially declining and then increasing at higher altitudes. KRE in CJ showed a steady decrease with increasing longitude and altitude, while no clear trend was observed along the latitudinal gradient. Overall, nutrient resorption efficiencies for N, P, and K in CJ exhibited a general decreasing trend with increasing longitude and a tendency to increase with latitude.

**FIGURE 7:**
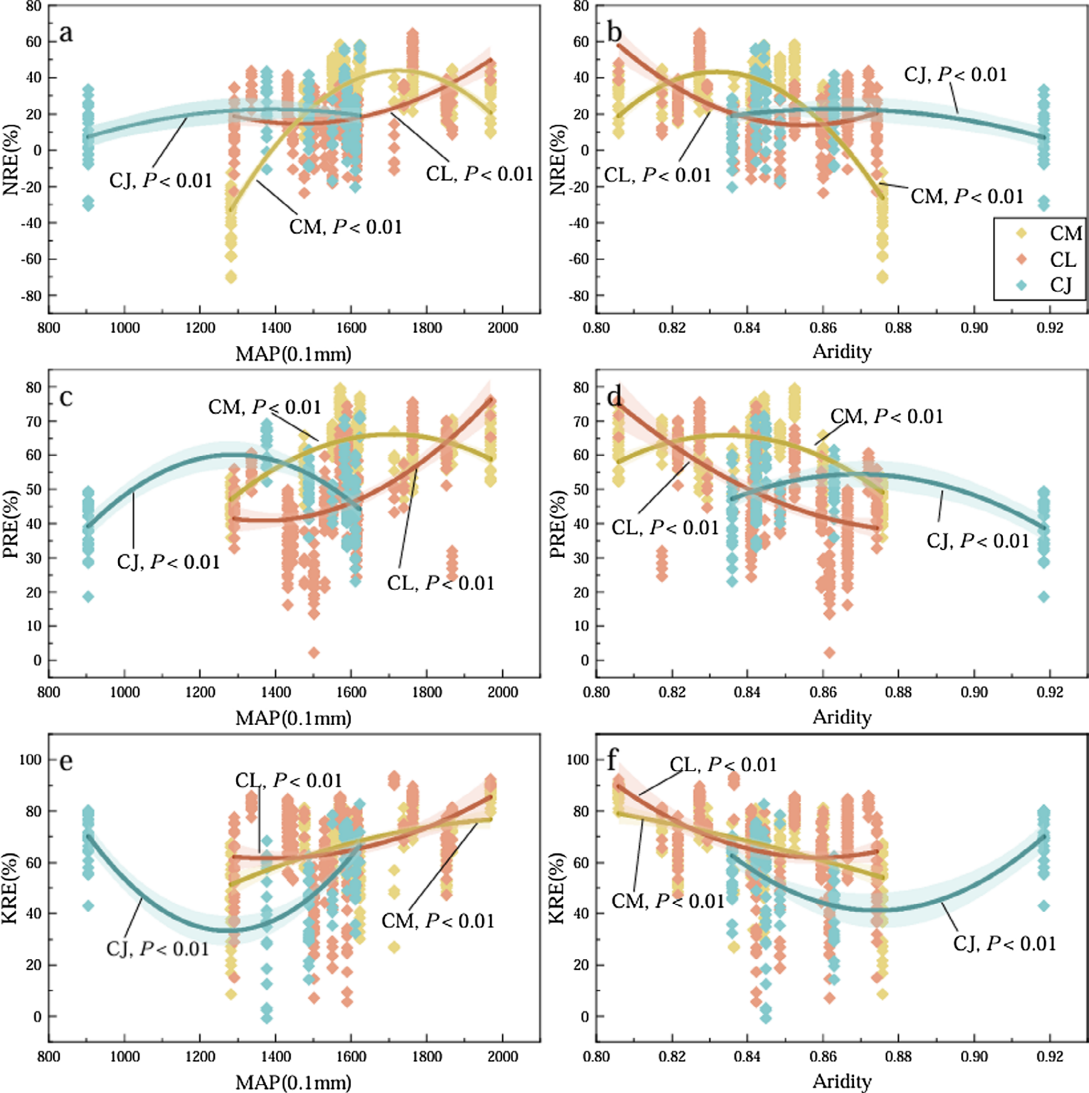
Change patterns of NRE, PRE, and KRE of ABs of three *Calligonum* species along with MAP and Aridity. CJ, *C. junceum* ; CL, *C. leucocladum* ; CM, *C. mongolicum* .

The variation patterns of NuRE in ABs of the three *Calligonum* species in response to MAP were generally opposite to those observed along the aridity gradient (Figure 7). For CM, both NRE and PRE increased initially and then declined with increasing MAP (or decreasing aridity), showing an overall upward trend, while KRE increased progressively across the MAP gradient. For CL, the resorption efficiencies of N, P, and K all increased consistently with increasing MAP (or decreasing aridity). For CJ, NRE exhibited a relatively weak response to MAP, with a slight upward trend. By contrast, KRE showed a unimodal response—decreasing at first and then increasing as MAP rose (or aridity declined).

**FIGURE 8:**
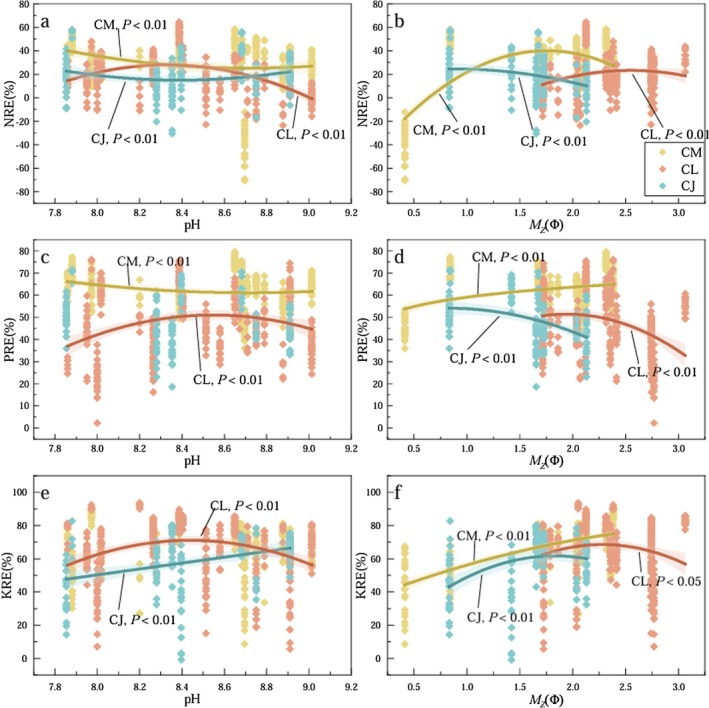
Change patterns of NRE, PRE, and KRE of ABs of three *Calligonum* species along with soil pH and sand particle size (M_Z_—Φ). CJ, *C.* junceum; CL, *C. leucocladum* ; CM, *C. mongolicum* .

As soil pH shifted from neutral to alkaline, CM showed a gradual decrease in both NRE and PRE, while the relationship between KRE and soil pH was not significant (Figure 8). For CL, the resorption efficiencies for N, P, and K followed a unimodal pattern, increasing first and then decreasing, with a turning point around pH 8.4. For CJ, NRE showed little variation with soil pH, PRE had no significant relationship with pH, whereas KRE exhibited a gradual increase with rising pH. With decreasing soil particle size, CM displayed an initial increase in NRE followed by a slight decline, while PRE and KRE showed consistent increasing trends (Figure 8). For CL, nutrient resorption efficiencies followed downward‐opening quadratic curves. As soil particles became finer, NRE generally increased, PRE gradually decreased, and KRE first increased then declined, though the overall variation in KRE was relatively minor. For CJ, NRE and PRE decreased with finer soil texture, whereas KRE increased overall.

### 3.4 Factors Influencing Nutrient Resorption Variability in Three *Calligonum* Species

Climatic factors were most strongly correlated with NRE, PRE, and KRE in CM, with 17 of 18 cases showing significant relationships (Figure S1). In CL, 9 of 18 cases were significant, while CJ showed significant correlations in 15 of 18 cases. Overall, with the exception of KRE in CJ—which increased under warmer and drier conditions (e.g., higher aridity and solar radiation, lower MAP)—the nutrient resorption efficiencies in CM and CL, as well as NRE and PRE in CJ, tended to decrease under high‐temperature, arid environments. Among the 11 soil variables examined, CM showed significant correlations in 29 of 33 cases, CL in 27 of 33 cases, and CJ in only 22 of 33 cases (Figure S2). The effects of specific soil factors—such as SWC, EC, MZ, TK, AN—on NuREs varied across species. By contrast, soil organic carbon (SOC), TN, TP were generally negatively correlated with NRE, PRE, and KRE in all three species, suggesting that under relatively nutrient‐rich soil conditions, plants reduced nutrient resorption from senescing tissues. Across all three species, NuREs were significantly and positively correlated with the nutrient concentrations of corresponding green ABs and negatively correlated with those of litter (Figure S3). This indicates that nutrient resorption in *Calligonum* species is jointly regulated by nutrient availability in both green and senesced tissues. Furthermore, NuREs in the three species exhibited significant relationships with geographic variables (longitude, latitude, and altitude) in nearly all cases. Together, these results suggest that geographic location, soil properties, climate, and intrinsic plant nutrient status jointly shape the nutrient resorption processes in the ABs of the three *Calligonum* species.

**FIGURE 9:**
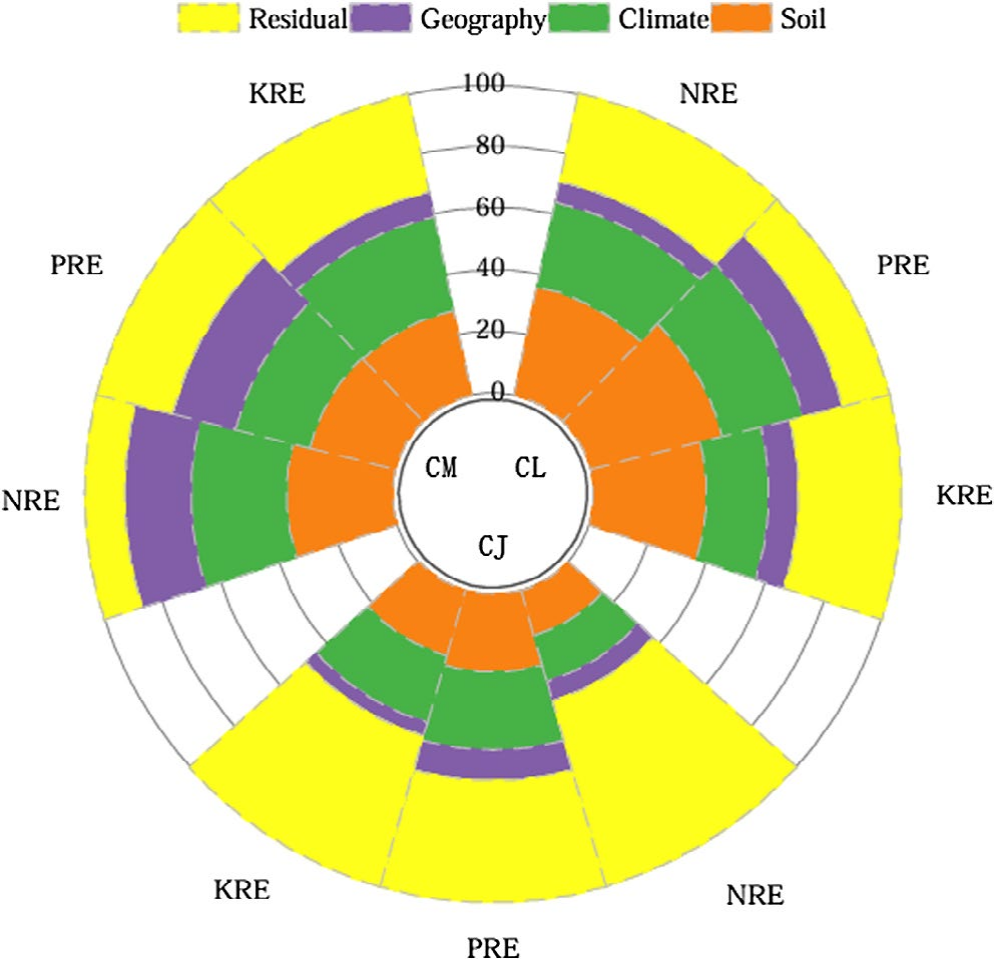
Variation partitioning of soil, climatic, and geographic factors on NuREs of three *Calligonum* species: CJ, *C.* junceum; CL, *C. leucocladum* ; CM, *C. mongolicum* .

Variance partitioning results (Figure 9) showed that soil, climate, and geographic factors collectively explained more than 65% of the variation in NuREs (NRE, PRE, and KRE) in both CM and CL. For CM (NRE and PRE) and CL (NRE, PRE, and KRE), soil factors accounted for the largest proportion of explained variance, followed by climate and geographic variables (soil > climate > geography). By contrast, for CJ, climate was the primary driver of NuRE variation, followed by soil and then geographic factors (climate > soil > geography). Hierarchical partitioning results (Figures S4 and S5) further indicated that, for CM, the largest proportion of explained variance in NRE and PRE was attributed to the combined effect of soil, climate, and geographic factors, while the variation in KRE was mainly explained by the joint effect of soil and climate. For CL, the unique effect of soil contributed most to the variation in NRE and PRE, whereas KRE variation was best explained by the combined effect of soil and climate. In CJ, the variation in NuREs was primarily driven by the shared effects of climate, soil, and geography, or by the joint effect of climate and soil, while the unique contributions of individual environmental factors were minimal.

## 4 Discussion

### 4.1 Characteristics and Interrelationships of NRE, PRE, and KRE in *Calligonum* ABs

Nutrient resorption is a key nutrient‐conservation strategy that facilitates internal nutrient cycling in plants. By reallocating nutrients from senescing tissues to growing organs before leaf or branch abscission, plants can reduce their dependence on soil nutrient availability (Jiang et al. 2019; Aerts and Chapin 2000). In this study, the NuRE of *Calligonum* species differed from global averages, with mean resorption efficiencies for N, P, and K all falling below the global values (62.1% for N, 64.9% for P, and 70.1% for K) (Vergutz et al. 2012). This global dataset includes Poaceae species, which are known to exhibit significantly higher resorption efficiencies than evergreen and deciduous woody plants (Vergutz et al. 2012). When compared with mean values reported for desert shrubs in Xinjiang (NRE: 51.0%, PRE: 54.9%, KRE: 46.3%) (Luo et al. 2020). *Calligonum* species showed lower NRE but relatively higher PRE and KRE. In karst ecosystems, plants tend to exhibit similarly low NRE (34.6%) but moderate to high PRE (48.4%) and KRE (63.2%), consistent with our findings. This pattern is often attributed to N‐rich but P‐deficient soils in karst regions (Liu et al. 2014). However, findings from a fixed‐site study in the Junggar Desert of northern Xinjiang reported a contrasting resorption pattern in CM, with PRE > NRE > KRE (Luo et al. 2020), which differs from the results observed in our study. It is worth noting that their conclusions were based on only two widely separated sampling sites and a limited sample size—approximately 122 samples from nine species collected during a single field campaign. By contrast, our study incorporated 17 CM sampling sites and a total of 420 samples collected in a standardized manner, providing a more representative and robust assessment of patterns. Therefore, discrepancies in sample size and spatial coverage are likely major contributors to the divergent findings and may limit the generalizability of the previous study's conclusions regarding CM.

According to previous studies, nutrient stoichiometric ratios in terrestrial vascular plants can serve as indicators of nutrient limitation types (Koerselman and Meuleman 1996; Olde Venterink et al. 2003; Guo et al. 2022). Based on earlier findings, the stoichiometric ratios in CM—with N:P < 14, N:K < 2.1, and K:P > 3.4—suggest that this species is primarily limited by N, or by P and N co‐limitation, while K is unlikely to be limiting (Luo et al. 2020). However, these interpretations appear inconsistent with our NuRE results. Across all three *Calligonum* species, NRE was relatively low, while PRE and KRE were much higher. This pattern suggests that P may be the more limiting nutrient, and K limitation may also occur, given the high KRE (Tian et al. 2021; Han et al. 2013). Although total nitrogen levels in the Gurbantunggut Desert are generally low, the availability of extractable (i.e., plant‐available) N in soils is relatively high (Tao et al. 2016). In addition, *Calligonum* species are known to form symbiotic associations with N‐fixing rhizobia (Feng et al. 2008), which may reduce the necessity for high internal N resorption. Supporting this, previous studies have shown that plants with lower NRE may enhance N mineralization, thereby increasing the availability of nitrogen in nutrient‐poor soils (Carrera et al. 2000). P limitation is common in arid ecosystems due to the slow biogeochemical cycling of P, which is primarily derived from long‐term rock weathering and dissolution processes (Wang et al. 2021; Gao et al. 2019). This may explain the consistently high PRE observed in all three species. K, on the other hand, is closely related to plant stress resistance, particularly in cold environments. The high KRE observed in *Calligonum* species may reflect a physiological demand for K to support overwintering and cold resistance. According to the Nutrient‐Limited Operational Strategies, plants tend to resorb the most limiting nutrients to a greater extent (Rajpurohit and Eiteman 2022). However, in this study, the nutrient resorption patterns of *Calligonum* species in the Junggar Desert did not align strictly with the Nutrient‐Limited Operational Strategies, which posits that plants preferentially resorb N in N‐restricted ecosystems (NRE > PRE) and resorb P in P‐restricted ecosystems (PRE > NRE) (Rajpurohit and Eiteman 2022). These findings suggest that while nutrient stoichiometry provides useful insights into potential nutrient limitations, it may not fully capture the physiological and ecological mechanisms that drive nutrient resorption in desert shrubs under extreme environmental conditions.

In all three *Calligonum* species, NRE–PRE exhibited allometric relationships with scaling exponents *α* > 1, indicating that the rate of N resorption increased more rapidly than that of P. Among the three species, CJ showed the highest scaling exponent, followed by CM and CL, suggesting that intra‐specific variation in N resorption was greater relative to P of CJ than in the other species. In CL, NRE was faster than both P and K resorption, and its PRE–KRE relationship followed an approximately isometric pattern, indicating a stable balance between the two nutrients. These patterns suggest that *Calligonum* species maintain more consistent PRE and KRE, potentially due to their generally higher PRE and KRE and comparatively lower NRE (Han et al. 2013).

The observed allometric relationships provide empirical support for the idea that nutrient resorption in *Calligonum* species conforms well to the Power Law of Nutrient‐to‐Nutrient Scaling, in line with the allometric principle widely observed in plant functional traits (Nikla 2006; He et al. 2020). According to this principle, nutrient concentrations in plant tissues of *Calligonum* species scale with one another in power‐function relationships, where the scaling exponent (*α*) may shift in response to both biotic and abiotic factors.

In addition, nutrient concentrations in mature ABs and litters have significant impacts on NuREs. In this study, all three species showed significant positive correlations between the NuREs and nutrient concentrations in summer ABs, while negative correlations were observed with nutrient concentrations in litters. This indicates that nutrient resorption in *Calligonum* is jointly regulated by nutrient status in both summer ABs and senesced tissues. Similar patterns have been reported in previous studies (Zhang et al. 2015; Zhou et al. 2016; Chen et al. 2015). It has been found that NRE is significantly positively correlated with nutrient concentrations in green leaves during the growing season and obviously negatively correlated with those in litters (Zhou et al. 2016); PRE is markedly negatively correlated with nutrient concentrations in litters; and KRE is significantly positively correlated with nutrient concentrations in green leaves and negatively correlated with those in litters. However, other studies have reported that NRE is either not significantly correlated or even negatively correlated with leaf N concentrations during the growing season, which contrasts with the results of this study (Liu et al. 2014; See et al. 2015). In a study conducted in the karst regions of southwestern China, it was reported that NuREs increased as nutrient concentrations in green leaves declined during the summer. Therefore, findings from different regions or plant functional types do not exhibit a consistent pattern, and there is no fixed relationship between NuREs and leaf nutrient concentrations.

### 4.2 Environmental Variation Patterns of N, P, and K Resorption in *Calligonum* ABs

Latitude and longitude are integrative proxies that reflect combined spatial gradients in climate and soil conditions. In this study, the NuRE of CM and CJ both showed a declining trend with increasing longitude. This pattern may be attributed to relatively higher soil nutrient availability in the eastern margin of the Junggar Desert. Previous studies have demonstrated a significant positive correlation between soil N content and longitude in this region (Tao et al. 2016). In this desert, the distribution of biological soil crusts and ephemeral plants—both of which play crucial roles in stabilizing sand surfaces and enhancing soil fertility—is largely restricted to the central, eastern, and southern parts of the desert, while being scarce in the west (Zhang et al. 2010; Duan et al. 2017). With increasing latitude, NRE and PRE of *Calligonum* species tended to increase, while MAT decreased. This trend is consistent with the findings from Vergutz et al. (2012). Although temperature variation across the study area is relatively modest, heat stress can accelerate leaf senescence, and K plays a key role in mitigating heat‐induced oxidative damage by regulating reactive oxygen species (Johnson et al. 2022; Martineau et al. 2017). In CL, KRE declined with increasing latitude, as did the K concentrations in both green ABs and litter. These results suggest that CL exhibits a degree of latitudinal sensitivity in K utilization, potentially due to a lower capacity for K uptake compared to CM and CJ. Previous research has shown that deciduous species often reallocate nutrients through strategic trade‐offs toward the end of the growing season to maintain internal nutrient balance (Du et al. 2017). Therefore, seasonal variation in nutrient concentration of ABs, as well as the nutrient status of older tissues, may be critical for understanding nutrient resorption and internal nutrient regulation in *Calligonum* species.

Previous studies have indicated that MAP significantly influences NRE in plant leaves (Jiang et al. 2019). For *Calligonum* species, NuREs generally declined with increasing aridity (i.e., decreasing MAP), indicating a positive correlation between nutrient resorption and annual precipitation. In CJ, both PRE and KRE exhibited quadratic relationships with aridity, which may be partly attributed to the lack of sampling sites in areas with intermediate aridity levels. When comparing across different spatial scales, the relationship between NRE and MAP observed in this study is consistent with previous findings. However, the pattern of PRE deviates from some earlier studies (Vergutz et al. 2012; Yuan and Chen 2008; Lynch and Clair 2004; Tang et al. 2013). For instance, a large‐scale study on woody plants in northern China found that aridity had no significant effect on NRE, PRE, or KRE (Lynch and Clair 2004), likely due to the broader range of aridity levels included. By contrast, within the narrower aridity gradient of the present study, aridity emerged as a key environmental constraint on nutrient resorption in *Calligonum* species. These findings support the Climate‐Driven Hypothesis (Yang et al. 2025), which posits that NuRE is influenced by climatic variables such as temperature and precipitation. Specifically, NRE tends to decrease with increasing temperature, while PRE increases with both temperature and precipitation.

In nutrient‐poor desert environments, where litter decomposition and subsequent nutrient release into the soil may be limited, plants often rely more heavily on internal nutrient resorption to meet their nutritional demands (Drenovsky and Richards 2006; Huang et al. 2016). In the present study, soil pH had a relatively minor overall effect on NuREs. For CL, nutrient resorption was highest under mildly alkaline conditions, suggesting that CL may enhance NuRE as an adaptive strategy to acquire nutrients under weak alkalinity. However, under more strongly alkaline conditions, nutrient resorption in CL declined, which may be related to increased water stress leading to premature tissue senescence (Huang et al. 2016). Soil particle size (M_Z_) showed a stronger correlation with nutrient resorption in *Calligonum* species, though the nature and strength of this relationship varied across species and nutrient types.

### 4.3 Environmental Drivers of N, P, and K Resorption Efficiencies in *Calligonum* ABs

The NRE, PRE, and KRE in *Calligonum* species were significantly positively correlated with each other, indicating that these three nutrient resorption processes are mutually reinforcing and may be under coordinated physiological regulation. At the global scale, studies have shown that climate zones significantly influence nutrient resorption patterns across plant functional types. For instance, MAP and latitude have been reported to strongly affect NRE, whereas the key ecological drivers regulating NRE and PRE differ (Jiang et al. 2019). However, at smaller spatial scales, the influence of climate on NRE tends to be weaker or inconsistent (Achat et al. 2018), highlighting that the relationship between nutrient resorption and climate varies across regions, scales, and species (Reich and Oleksyn 2004). In our study, most climatic variables (except MAP) were negatively correlated with NuREs in CL. For CM and CJ, NRE and PRE showed similar correlations with climatic factors, while KRE exhibited the opposite trend. Interestingly, MAP was positively correlated with nutrient resorption efficiencies in all species (except for KRE of CJ), which contrasts with findings from global‐scale analyses (Jiang et al. 2019). Moreover, nutrient resorption in *Calligonum* species was positively associated with nutrient concentrations in green ABs and negatively associated with those in senesced tissues. These results indicate that nutrient resorption is simultaneously regulated by the nutrient status of both current‐season growth and litters, reflecting a complex internal‐external nutrient coordination strategy.

Geographic, climatic, and soil variables together explained more than 65% of the variation in NuREs for CM and CL, and over 37% for CJ. However, the dominant environmental drivers varied among species and nutrients. For CM, soil factors were the primary drivers of NRE and PRE, whereas climate was the main factor influencing KRE. In CL, soil variables contributed the most to the variation in all three NuREs, with their unique effects accounting for the largest proportion of explained variance. By contrast, climate emerged as the dominant factor regulating nutrient resorption in CJ. These results highlight that soil factors play a primary role in shaping nutrient resorption patterns in CM and CL, whereas CJ appears to be more influenced by climatic conditions. Soil water availability, in particular, plays a crucial role in determining nutrient availability within the soil matrix and also affects litter decomposition, which in turn influences nutrient cycling processes (Liu et al. 2006). Long‐term studies have shown that nutrient mineralization rates largely determine soil nutrient levels, and plant species, through their environmental adaptations, occupy distinct biogeochemical niches (Tian et al. 2021), which ultimately influence their nutrient resorption capacities (Aerts and Chapin 2000; Killingbeck 1996). Soil and climate often interactively affect plant physiology. For instance, reduced precipitation can lead to lower soil moisture and exacerbate plant water stress. In arid conditions, accelerated tissue senescence may prematurely interrupt nutrient resorption processes (Lu et al. 2018; Rivero et al. 2007; Killingbeck 1996). Compared with CM and CL, nutrient resorption in CJ appears to be less tightly linked to soil conditions, suggesting that CJ may occupy a different biogeochemical niche and adopt alternative adaptive strategies (Sardans and Peñuelas 2014). It is also important to recognize that the unexplained variation in nutrient resorption points to the potential influence of other environmental variables not included in the current analysis. These may include internal factors such as plant nutrient status, physiological traits, or genetic characteristics. This highlights a promising direction for future research.

All in all, there is no universal superiority or disadvantage of NuRE values for plants, and it needs to be dynamically and comprehensively weighed according to plant functional type, habitat nutrient status, and environmental stress intensity. The goal‐oriented researches (such as ecological restoration and carbon sink improvement) need to be targeted to regulate NuREs to achieve optimal resource allocation. The differences in the nutrient resorption strategies of the three species do not determine the success or failure of their survival strategies, but can deepen our understanding of their environmental adaptation.

## 5 Conclusion

In this study, we systematically analyzed the variation patterns and environmental drivers of NuREs (NRE, PRE, and KRE) in ABs of three *Calligonum* species representing different taxonomic sections across the Junggar Desert in northwestern China. Overall, *Calligonum* species exhibited the trend KRE > NRE, with all three NuREs falling below global averages. Among species, NRE and PRE were highest in CM, while CJ had the lowest KRE, reflecting clear interspecific differences. All three species demonstrated strong scaling relationships of NRE–PRE (slope > 2), indicating that NRE increases faster than PRE, although this does not necessarily correspond to higher NRE. In CJ, the scaling exponent of PRE–KRE was negative, suggesting a significant trade‐off between the two processes. Overall, nutrient resorption in *Calligonum* species largely conformed to the Power Law of Nutrient‐to‐Nutrient Scaling. NuREs were jointly regulated by nutrient concentrations in both green ABs (positively) and litters (negatively). The three NuREs within each species tended to respond similarly to geographic, climatic, and soil gradients; considerable interspecific variation was observed. Soil and climate emerged as the dominant environmental factors influencing NuREs of the three species, though the relative contributions and interaction effects varied by species and nutrient type. These findings are broadly consistent with the climate‐driven hypothesis, which emphasizes the role of temperature and precipitation in regulating nutrient resorption, but deviate from the predictions of the Nutrient‐Limited Operational Strategies. In a word, all of the NuRE characteristics presented marked interspecific differences, although they belonged to the same genus. These findings provide important insights into the nutrient utilization strategies and survival mechanisms of different *Calligonum* species and assimilating‐branch shrubs. They also offer valuable data support for the further exploration and sustainable use of these valuable plant resources.

This study is primarily based on measured data from the Junggar Desert. However, since the sampling range is limited to a single geographical unit, whether these specific environmental driving relationships can be directly applied to other habitats still needs further verification.

## Author Contributions

**Su‐Su Wei:** investigation (equal), methodology (equal), visualization (equal), writing – original draft (equal). **Yuan‐Yuan Zhang:** data curation (equal), investigation (equal), methodology (equal), visualization (equal), writing – original draft (equal). **Xin‐Yue Jin:** data curation (equal), investigation (equal), methodology (equal), visualization (equal), writing – original draft (equal). **Yue Zhang:** investigation (equal), visualization (equal). **Xiao‐Bing Zhou:** conceptualization (equal), methodology (equal), writing – review and editing (equal). **Gang Huang:** conceptualization (equal), funding acquisition (equal), writing – review and editing (equal). **Ye Tao:** conceptualization (equal), funding acquisition (equal), investigation (equal), methodology (equal), project administration (equal), resources (equal), writing – review and editing (equal).

## Funding

This work was supported by the Natural Science Foundation of Xinjiang Uygur Autonomous Region, Grant No. 2022D01A346; the Western Light talent cultivation program of Chinese Academy of Sciences, Grant No. 2022‐XBQNXZ‐006; the Leading Talents in Sci‐Technological Innovation Project of “Tianshan Talent” Training Plan of Xinjiang Uygur Autonomous Region, Grant No. 2022TSYCLJ0058; National Natural Science Foundation of China, Grant No. 42171070.

## Conflicts of Interest

The authors declare no conflicts of interest.

## Supporting information


**Data S1:** ece372853‐sup‐0001‐FigureS1‐S5.pdf.

## Data Availability

The relevant data of this study have been publicly stored at DRYAD (https://doi.org/10.5061/dryad.tx95x6bb6).
