# Correction to “Divergent Nutrient Resorption Strategies in C4 Desert Shrubs: Stoichiometric Evidence From Assimilative Branches”

**DOI:** 10.1002/ece3.73163

**Published:** 2026-02-26

Wei, S.‐S. , Y.‐Y. Zhang , X.‐Y. Jin , Y. Zhang , X.B. Zhou , G. Huang , Y. Tao 2026. “Divergent Nutrient Resorption Strategies in C4 Desert Shrubs: Stoichiometric Evidence From Assimilative Branches.” Ecology and Evolution 16, no. 1: e72853. 10.1002/ece3.72853.41531901 PMC12793778

In the published version of the above article, errors occurred in the author–affiliation numbering and funding information.

Except for the first author, the superscript affiliation numbers for the other authors were incorrectly assigned. The correct author–affiliation correspondence is as follows:

Su‐Su Wei^1,2,3,4^, Yuan‐Yuan Zhang^2,3,4^, Xin‐Yue Jin^2,3,4^, Yue Zhang^2,3,4^, Xiao‐Bing Zhou^2,3,4^, Gang Huang^1^, Ye Tao^2,3,4^

The corresponding affiliations are:

^1^College of Life Sciences, Shihezi University, Shihezi, China

^2^State Key Laboratory of Ecological Safety and Sustainable Development in Arid Lands, Xinjiang Institute of Ecology and Geography, Chinese Academy of Sciences, Xinjiang, China

^3^China‐Tajikistan Belt and Road Joint Laboratory on Biodiversity Conservation and Sustainable Use, Xinjiang Institute of Ecology and Geography, Chinese Academy of Sciences, Urumqi, China

^4^Xinjiang Key Laboratory of Biodiversity Conservation and Application in Arid Lands, Xinjiang Institute of Ecology and Geography, Chinese Academy of Sciences, Xinjiang, China

In addition, the funding information provided in the “Funding” section was incorrect. The correct funding statement is as follows: “This work was financially supported by the National Natural Science Foundation of China (Grant No. 42171070), the “Western Light” Talent Cultivation Program of the Chinese Academy of Sciences (Grant No. 2022‐XBQNXZ006), and the Leading Talents in the Sci‐Technological Innovation Project of “Tianshan Talent” Training Plan of Xinjiang Uygur Autonomous Region (Grant No. 2022TSYCLJ0058).”

We apologize for this error.

