# Supplementary material for: Divergent Nutrient Resorption Strategies in C4 Desert Shrubs: Stoichiometric Evidence From Assimilative Branches
Source: Ecol Evol. 2026 Jan 12;16(1):e72853. doi: 10.1002/ece3.72853 (PMC12793778; doi:10.1002/ece3.72853)
Supplement: Supplementary file 1 — Data S1: ece372853‐sup‐0001‐FigureS1‐S5.pdf. [file ECE3-16-e72853-s001.pdf]

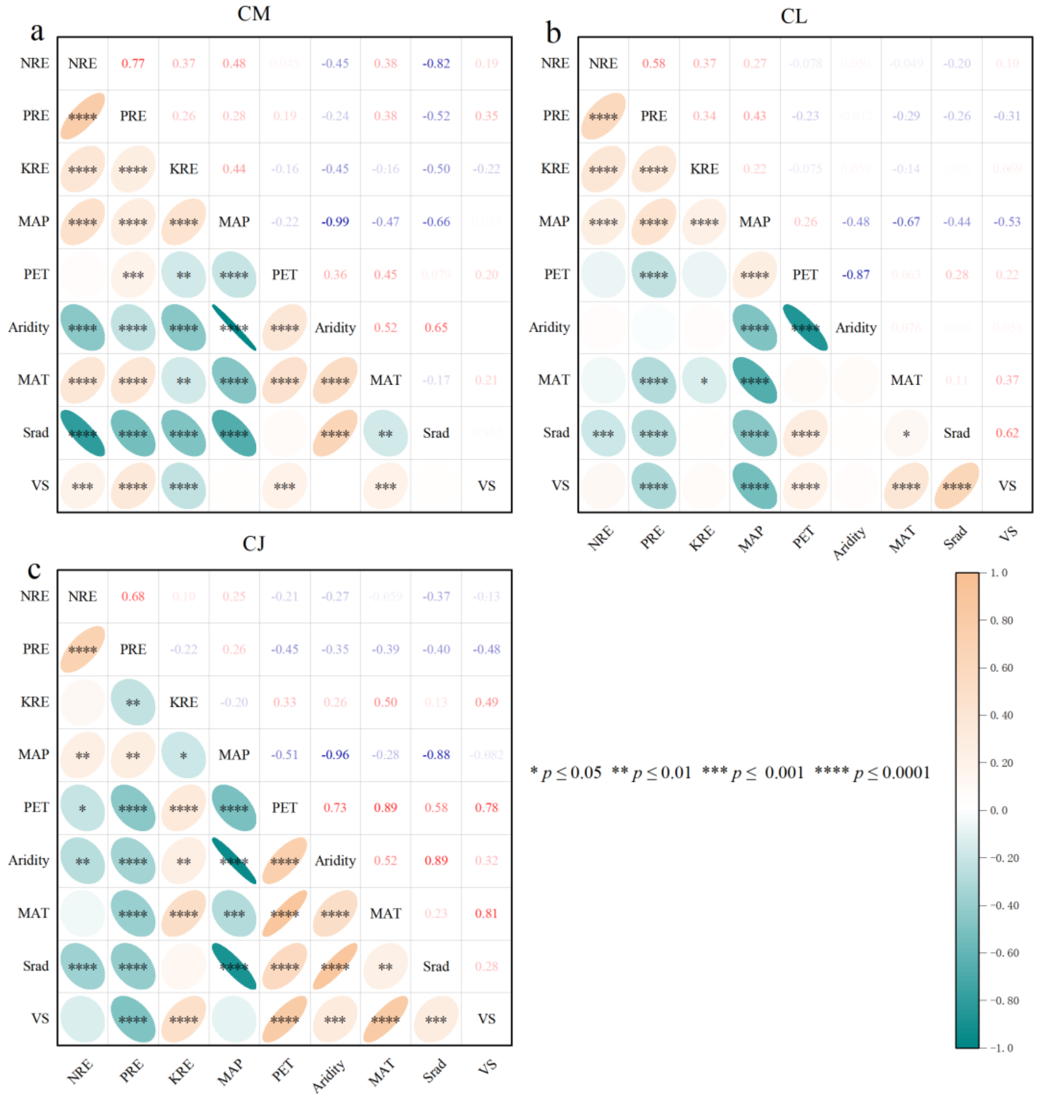

Fig. S1 Correlations between nutrient resorption efficiencies and climatic factors for three Calligonum species. CM: *C. mongolicum*; CL: *C. leucocladum*; CJ: *C. junceum.*

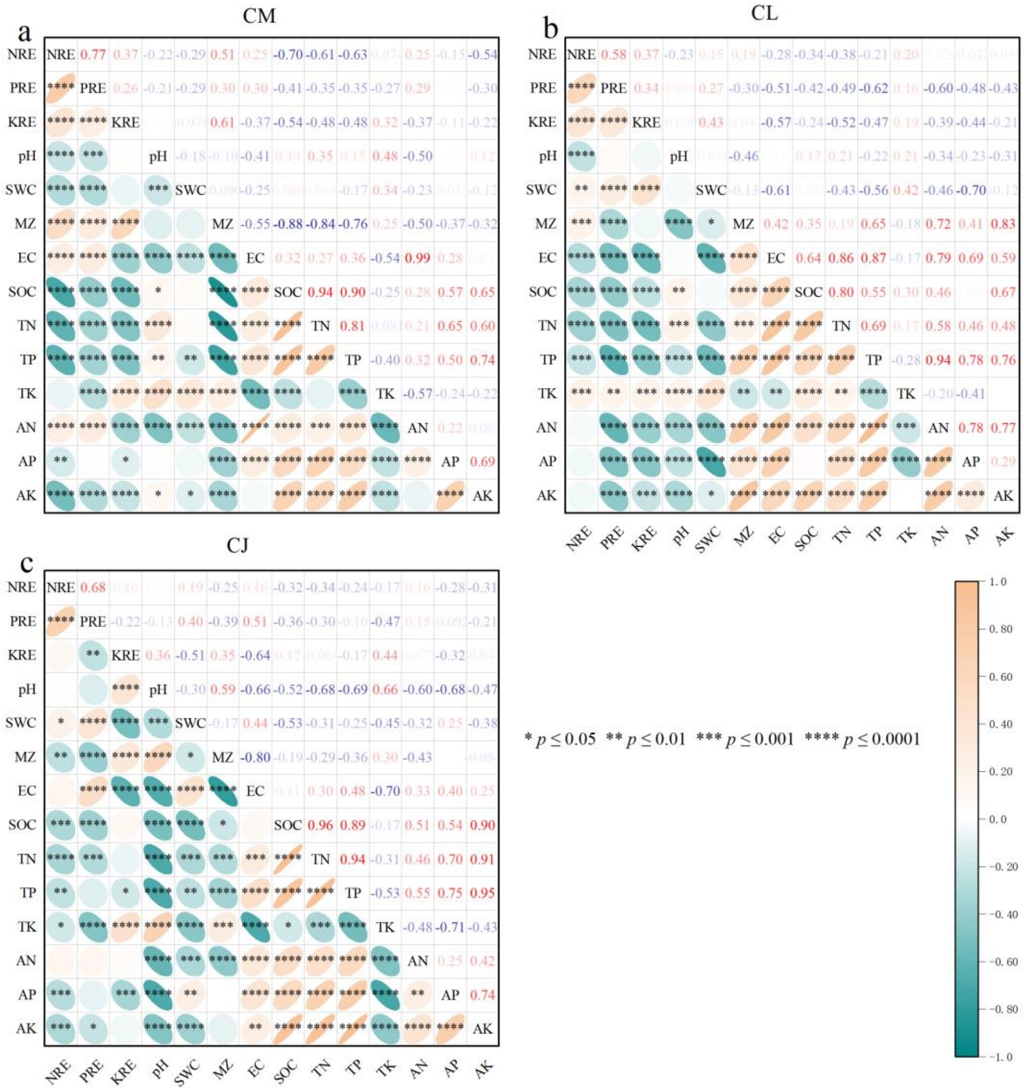

Fig. S2 Correlations between nutrient resorption efficiencies and soil factors for three *Calligonum* species. CM: *C. mongolicum*; CL: *C. leucocladum*; CJ: *C. junceum.*

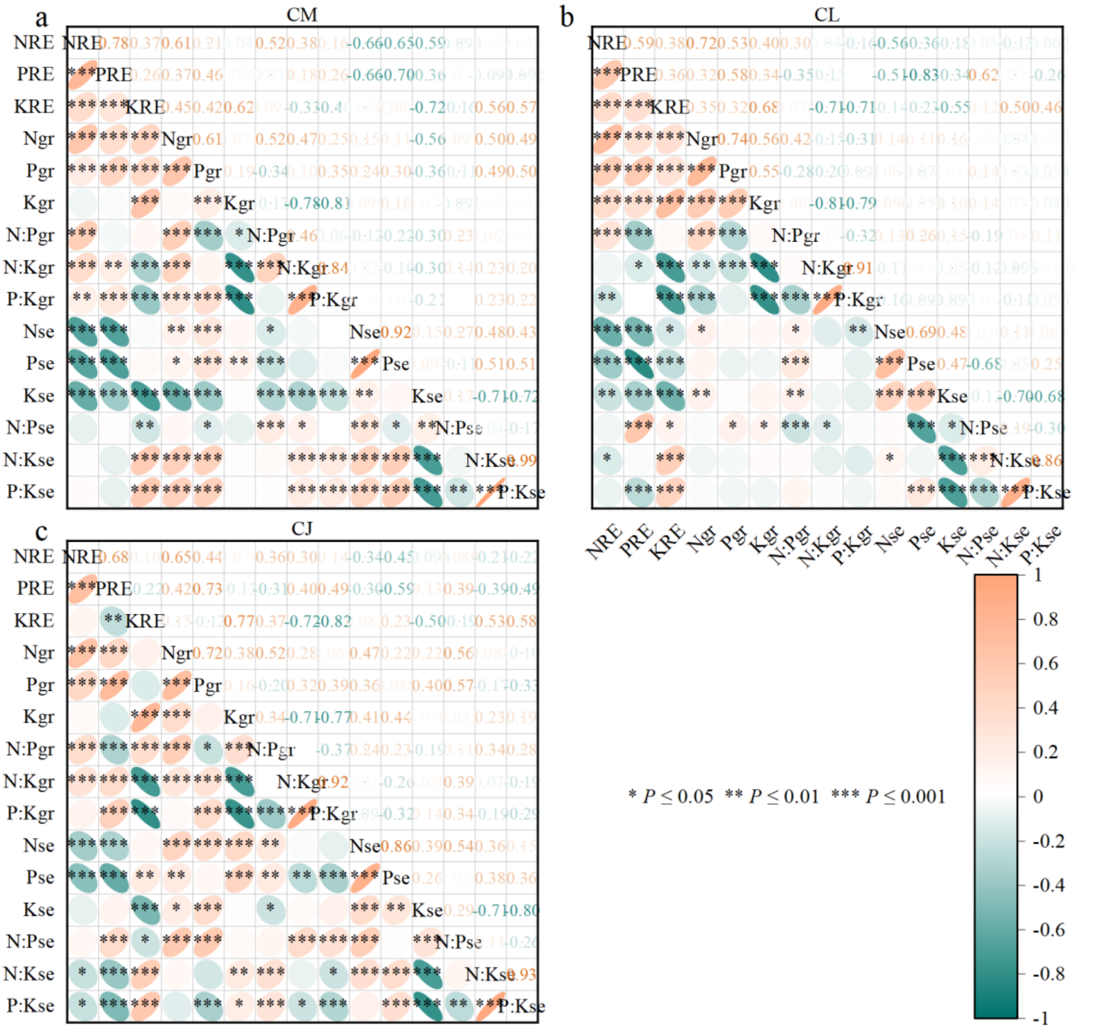

Fig. S3 Correlations between nutrient resorption efficiencies and nutrient status of green and senesced assimilative branches for three *Calligonum* species. CM: *C. mongolicum*; CL: *C. leucocladum;* CJ: *C. junceum.*

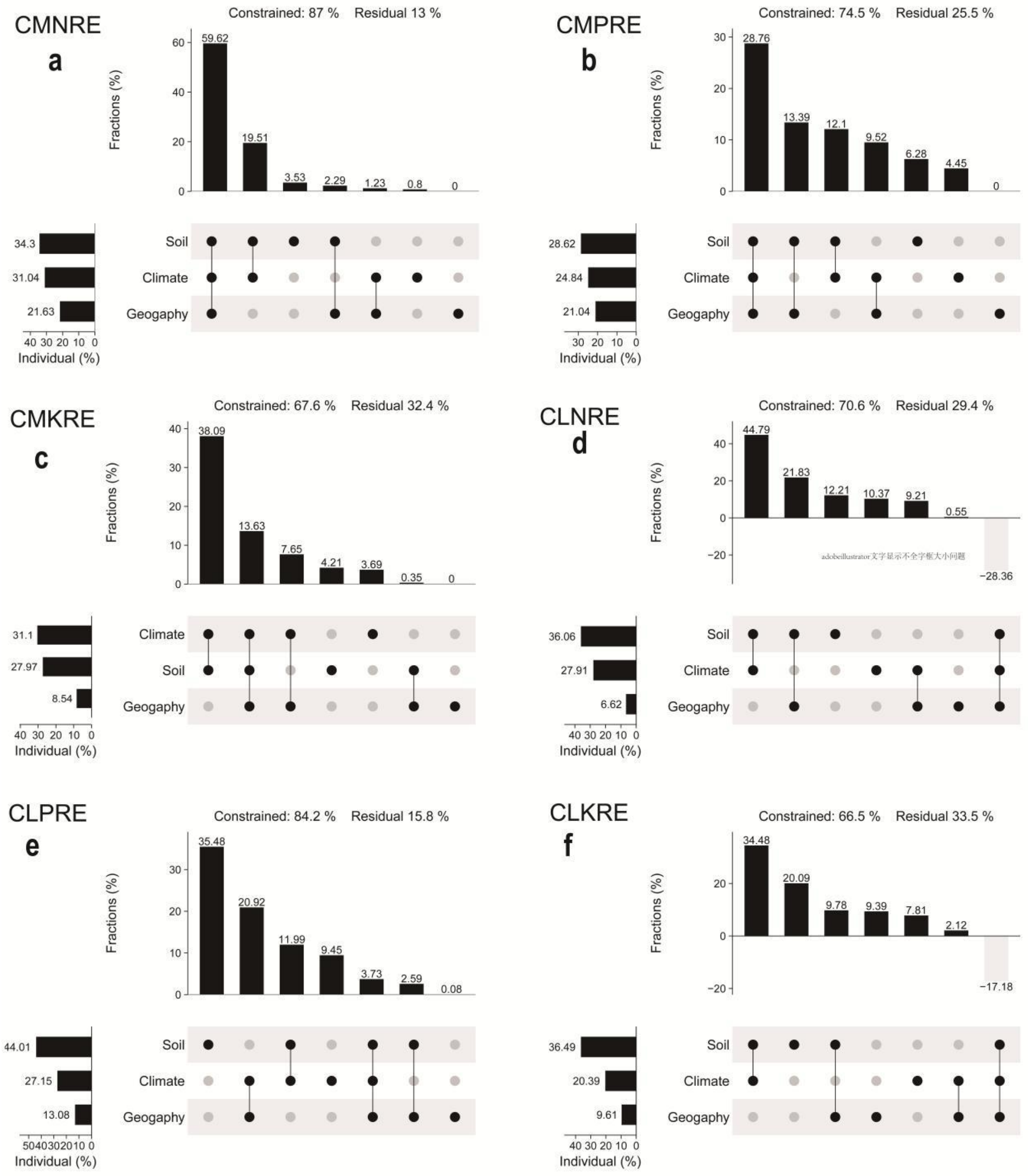

Fig. S4 Contributions of soil, climatic and geographic factors to nutrient resorption efficiencies of *C. mongolicum* and *C. leucocladum*

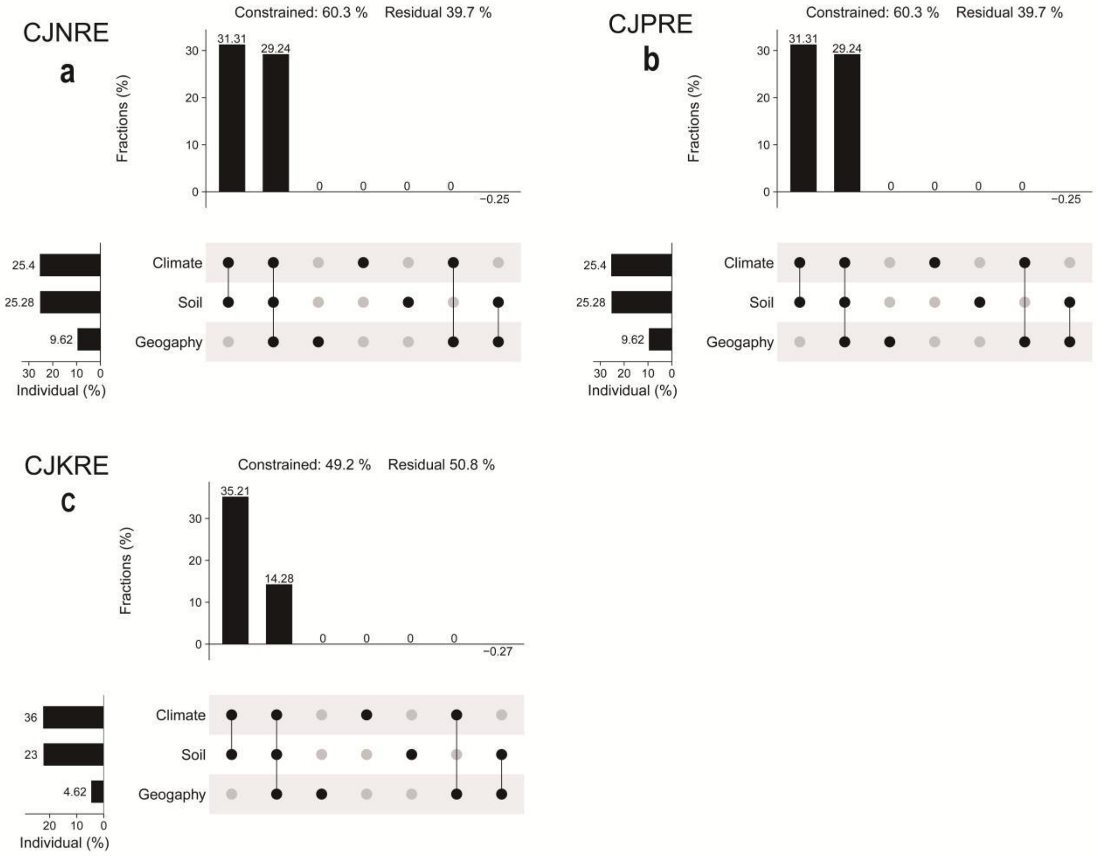

Fig. S5 Contributions of soil, climatic and geographic factors to nutrient resorption efficiencies of *C. junceum*
